# Transcriptional regulatory logic orchestrating lymphoid and myeloid cell fate decisions

**DOI:** 10.3389/fimmu.2025.1544483

**Published:** 2025-05-29

**Authors:** Rasoul Godini, Jingjing Yan, Michaël Chopin

**Affiliations:** ^1^ Development and Stem Cells Program, Monash Biomedicine Discovery Institute, Melbourne, VIC, Australia; ^2^ Department of Anatomy and Developmental Biology, Monash University, Melbourne, VIC, Australia; ^3^ Department of Biochemistry and Molecular Biology, Cancer and Immunity Programs, Monash University, Melbourne, VIC, Australia

**Keywords:** lymphoid cells, myeloid cells, transcriptional regulation, network analysis, dendritic cell

## Abstract

**Introduction:**

The differentiation of hematopoietic stem cells (HSCs) into diverse blood and immune cells is a complex, highly hierarchical process characterized by a series of tightly regulated steps. It involves a sequence of intermediate oligo-potent progenitors making successive binary decisions. This process gradually narrows down lineage possibilities until a final fate is reached. This step-wise process is tightly controlled by transcription factors (TFs) and their associated regulome, ultimately resulting in the differentiation of both lymphoid and myeloid compartments.

**Methods:**

We unravel the lineage-specific gene regulatory circuitry controlling the development of B cells, T cells, innate lymphoid cells (ILCs), and dendritic cells (DCs). We employ weighted gene co-expression network analysis to characterize gene modules associated with the lymphoid or myeloid cell fate, enabling the identification of lineage-restricted TFs based on their expression patterns.

**Results:**

By identifying TFs whose expression is subset-restricted or those with a broader expression in the hematopoietic compartment, we construct a regulatory logic that potentially controls the development of these key immune cells. Our results point to conserved regulatory elements between ILCs, natural killer cells, and DCs. This analysis unravels an intricate relationship between each cell type and how the expression of key TFs dictates lineage specificity. We particularly dissect the elements associated with conventional DCs and plasmacytoid DCs.

**Discussion:**

In conclusion, our findings shed new light on regulatory mechanisms controlling blood cell development and offer a blueprint that can be leveraged to better understand the molecular mechanisms underpinning blood cell development.

## Introduction

Hematopoiesis continuously generates billions of blood cells each day, encompassing various cell types with distinct functions essential for maintaining physiological homeostasis and immune competence. In adult mammals, hematopoiesis is driven by hematopoietic stem cells (HSCs) residing in the bone marrow. These HSCs undergo a series of tightly regulated maturation steps, giving rise to successive oligopotent progenitors that progressively lose multipotency to differentiate into a range of specialized mature blood cells (1). In brief, HSCs are categorized by their self-renewal and multipotency. Long-term HSCs (LTHSCs) have a prolonged self-renewal ability and give rise to short-term HSCs (STHSCs), which possess a more restricted self-renewal potential. STHSCs further differentiate into multipotent progenitors (MPPs), which lack the self-renewal ability but contribute to the generation of various blood cell lineages (1). MPPs subsequently differentiate into common myeloid progenitors (CMPs), producing erythrocytes, megakaryocytes, and monocytes (2), and common lymphoid progenitors (CLPs), which give rise to B cells, T cells, and natural killer (NK) cells (3–6).

This hierarchical process requires the dynamic regulation of TF networks that activate lineage-specific gene expression and restrict the differentiation options of hematopoietic progenitors (7–10). For example, *Runx1* and *Gfi1* are essential for the development of HSCs (11, 12), and *Runx1* continues to play a pivotal role in guiding dendritic cell (DC) development from myeloid progenitors (13). *Pax5* and *Ebf1* regulate B-cell development (14, 15), while PU.1 and IRF8 are instrumental for dictating dendritic cell commitment (16–19). Thus, the coordinated action of TFs is crucial for directing key regulatory nodes that instruct the emergence of diverse blood cell lineages.

The integration of transcriptome data across various blood cell types, along with chromatin profiling, has significantly advanced our understanding of the regulatory mechanisms governing hematopoiesis (20, 21). Adding to that, a comprehensive mapping of the cis-regulatory elements associated with the development of 86 immune cell populations is readily available (22). Moreover, the role of epigenetic modifications influencing chromatin dynamics during hematopoiesis has been studied (23, 24). Despite the availability of these resources, there is a critical need to construct detailed gene regulatory networks (GRNs) that elucidate the differentiation and the functional attributes of the different blood cells produced from HSCs. Such networks are essential for uncovering the overarching principles of gene regulation and the transcriptional circuitry that directs lineage decisions. This understanding will not only offer valuable insights into the process of blood cell formation but also pave the way for exploring novel approaches for *ex vivo* blood cell production.

Herein, we apply network analysis to the high-throughput transcriptome data of 48 cell types produced by Yoshida and colleagues (22) under the ImmGen project (www.immgen.org/). Through this analysis, we have identified lineage-specific transcription factors (TFs) and developed a network illustrating their interactions. Additionally, we introduce several novel transcription factors, ranked according to their significance and interaction strength. Finally, we validate the efficacy of our approach by identifying a novel transcription factor crucial in the decision-making process of a progenitor to differentiate into functionally distinct DC subsets.

## Materials and methods

### Data collection

In this study, we applied public-accessible datasets GSE109125 of transcriptome data of 127 populations and GSE100738 of ATAC-seq data of 86 primary cell types (22). The datasets were filtered for stem cells (including LTHSCs, STHSCs, MPP3, and MPP4), lymphoid cells [B cells, α/βT cells, and **i**nnate **l**ymphoid **c**ells (ILCs)], and DCs as a group differentiated from both myeloid and lymphoid cells. Thus, 91 samples of immune cells were used for further analysis (**Supplementary File 1**). For ATAC-seq data, we used only the corresponding cells to the transcriptome 39 sample of immune cells (**Supplementary File 2**). The ILC group comprises ILC2s, ILC3s, and natural killer cells. Additionally, the data were excluded if miss-annotated, and activated T cells and γδ T cells were excluded from our analysis. Each lineage encompasses various transitional states. Therefore, analyses were performed using different approaches, including i) lineage-wise, where cells are compared as lineages, and ii) subgroup-wise, which compares individual subgroups against each other. For the analysis of the ATAC-seq data, we only focus on the cis-regulatory regions associated with the genes of interest using open chromatin region (OCR) data.

### RNA analysis

RNA-seq analyses were performed using the edgeR package in R (25). Samples were grouped as cell lineages and compared to the stem cells group (including LTHSC, STHSCs, CLPs, and CMPs). A gene was included for the analysis if it had a minimum count of 10 in more than 70% of the samples and a total count of 100 across all the samples. The genes were significant if the log2-fold change was ±0.585 for TFs and ±1 for other genes. Only genes with a false discovery rate (FDR) ≤ 0.05 were considered significant. The specificity of genes to each cell group was calculated as:


$$Specificity=\;\frac{Average\;expression\;in\;cell\;group\;of\;interest}{Mean\;of\;average\;expression\;in\;all\;cell\;groups\;}$$

### Gene filtering variable genes and sample clustering

To study the distribution of data, samples were clustered using hierarchical clustering (method = “average”) and Principal Component Analysis (PCA) (26) using R. TFs were identified by comparing the gene list against 1,611 from AnimalTFDB 3.0 (27).

### Weighted gene co-expression network analysis

Weighted gene co-expression network analysis (WGCNA) was performed using the WGCNA package in R (28). Based on the clustering, samples were grouped into cell lineages and compared to identify co-expressed genes in each group. To analyze all the lymphocytes plus DCs, the network was constructed by a soft-power “16” and “signed” type, and modules were identified according to a minimum module size of “50” and a deep split of “2”. To analyze the DCs, we used a soft-power of “12” due to the smaller number of samples, and deep-split of “2”. The modules were selected based on high association with cell lineages for further analysis. The networks or modules were extracted with a threshold of correlation > 0.01 and thereafter analyzed and visualized using Cytoscape 3.8.2 (29). Through the experiments, genes of interest were filtered using in-house-developed R scripts.

### Gene regulatory network construction

The regulatory networks were constructed through two approaches. First, by applying the WGCNA results using only the association between TFs and other genes. This approach produces undirected networks of highly expressed genes in particular lineage/s. WGNCA regulatory networks were then combined with information from ChIP Enrichment Analysis from the ChEA database (30, 31) and RegNetwork database (32). Briefly, to obtain potential TF-gene interactions, we submitted a list of cell-specific differentially expressed genes (DEGs) (against stem cells) or DEGs present in a particular group of families or all cells, to the ENRICHR online tool (30). We compared the resulting potential regulators and kept those with a p-value ≤ 0.05 and those specifically expressed in the corresponding cell(s). Additionally, the expressed TFs, either DE-TFs or genes detected through WGCNA, were compared against RegNetwork data (32) to find potential interactions. The final network was constructed by combining all the networks (from WGCNA, ChEA2022, and RegNet), and removing duplicate interactions. We constructed a network of only TFs by presenting only TF-TF interactions and showing the centrality of TFs by adding the TF-gene interaction scale number as the size of the node. The network analysis and adding parameters, such as gene expression, gene connectivity within the network, and ATAC-seq data, were done using Cytoscape 3.8.2 (29).

### Gene Ontology enrichment

The DAVID database was used for the Gene Ontology (GO) enrichment analysis (33). The results with a p-value ≤ 0.05 and top matches were used for further analysis.

### *Bhlha15* cloning

A full-length coding sequence flanked by Xho1 sites of *Bhlha15* was amplified from cDNA generated from RNA isolated from wild-type (wt) splenic pDC. cDNA was cloned into a TOPO-TA cloning kit (Invitrogen) according to the manufacturer’s recommendations. *Bhlha15* cDNA was subsequently subcloned into pMSCV-iresGFP upstream of the IRES-GFP reporter gene.

### Cell culture

Isolated bone marrow cells were cultured in mouse tonicity RPMI-1640 supplemented with 10% heat-inactivated fetal calf serum, 2 mM l-Glutamine (Gibco), 50 µM 2-mercaptoethanol (Sigma-Aldrich), and 100 U/ml penicillin/streptomycin (Gibco). Furthermore, 1.5×10^6^ cells/ml were stimulated with 100 ng/ml of Flt3L (Peprotec) for up to 7 days.

### Retroviral infection

Retroviral supernatants were generated by transient transfection of 293T cells with plasmids encoding viral envelope proteins (pMD1-gag-pol and pCAG-Eco), and expression vectors encoding for pMSCV-iresGFP and pMSCV-Bhlha15-iresGFP using FuGeneHD (Promega). Retroviral supernatants were centrifuged onto RetroNectin (Takara)-coated plates for 45 min at 4,000 rpm at 4°C. Cells were then cultivated with the virus in the presence of 4 μg/ml polybrene (Sigma-Aldrich) for 12 hr. Then, 96 h after infection, cells were harvested and stained for flow cytometry analysis.

### Flow cytometry and antibodies

Single-cell suspensions were resuspended in PBS + 2 mM EDTA + 0.5% BSA (Sigma-Aldrich) and stained with the indicated antibodies at 4°C. All the analyses were performed on a FACSCanto (BD Biosciences) and data were processed using FlowJo. Antibodies against CD11c (N418), MHCII (M514.15.2), XCR1 (ZET), SiglecH (551), and CD11b (M1/70) were purchased from BioLegend. Propidium Iodide (Sigma) was used to exclude dead cells.

### Literature review

To identify known TFs involved in the development or functioning of lymphoid cells and DCs, we searched the literature with keywords, including the name of the TF of interest and the cell type in which we detected the TFs. We included information in two tables, one for known TFs referencing their PubMed ID (**Supplementary File 3**, **Supplementary Figure S1**) and a table showing TFs with no identified function in the corresponding cells. The TFs with no direct study or only having the gene expression in the publication were classified as unknown.

## Results

### High degree of expression similarities across cells of white blood cell lineages

**Figure 1:**
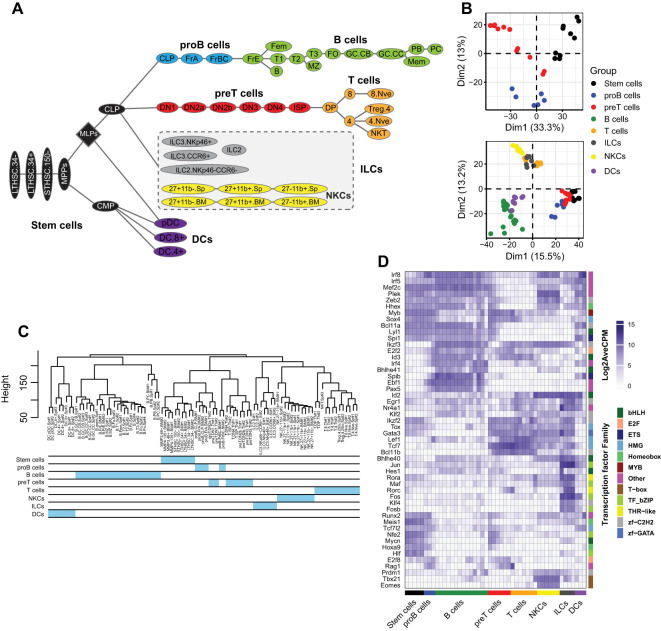
Lymphoid cell and DC lineage classification and gene expression. **(A)** The developmental tree of the lymphoid cells and DCs. The lineages and subset cell groups used in our study. We considered the NKC family a separate group of ILCs (with modification from (22)). **(B)** PCA plots of the top 3,000 most variable genes of all cell types, and the only stem cells to obtain higher resolution. **(C)** Hierarchical clustering of all cells. **(D)** Heatmap of the top 50 most variable TFs across all cell types. DCs, dendritic cells; ILCs, innate lymphoid cells; NKCs, natural killer cells.

The mammalian blood system consists of multiple lineages stemming from HSCs. To visually capture the development pathways and relationships between the different blood cell types, hematopoiesis is commonly depicted as a tree structure, reflecting the lineage tracing from a common progenitor. In this study, we leveraged publicly available resources to analyze the relationships between progenitors and mature blood cells, including B cells, T cells, innate lymphocytes, and dendritic cells (22) (**Figure 1A**). To identify gene signatures within each group, the cells were clustered into stem cells, pro-B cells, pre-T cells, B cells, T cells, ILCs, NKs, and DCs. We examined the clustering of the cells by analyzing transcriptome data using hierarchical clustering and PCA. The results of both methods showed that the members of each group were clustered together (**Figures 1B, C**). Notably, stem cells and proB and preT cells were in very close clusters, suggesting that even as they commit to B or T cell lineages, proB and preT cells retain a substantial level of stemness (**Figures 1B, C**). However, applying a finer resolution by conducting PCA exclusively on the stem cells and proB and preT cells reveals a broader distribution among these cells, thus highlighting sharp differences among these progenitors (**Figure 1B**). This analysis revealed that T cells, ILCs, and NK cells were closely grouped, demonstrating some degree of similarities between these cells (**Figure 1B**). Hierarchical clustering corroborated these findings, aligning closely with the PCA results (**Figure 1C**). These analyses showed that subsets within each family lineage exhibited a similar transcriptome that sets them apart from others. This fact, despite the fine differences within subgroups of cells, allows us to compare each subset as a group against stem cells or other lineages in further experiments (**Figure 1A**).

To identify regulatory elements specific to each lineage, we identified the top 50 most variable TFs across all the samples. Our results revealed that TFs were expressed either exclusively within a particular lineage or across multiple lineages (**Figure 1D**). For example, as expected, *Ebf1* and Pax5 were exclusively expressed in proB and B cells, whereas Spib also expressed DCs. In contrast, some TFs, such as *Ikzf3* and *E2f2*, were expressed across many cell types.The clustering of blood cells into distinct groups reflects the high transcriptomic similarity within each group and suggests specific regulatory mechanisms, which we revealed by identifying the most variable TFs across cell groups. However, focusing solely on the most variable genes provides an incomplete picture. To enhance our analysis, we performed WGCNA, which identified a broader set of group-specific TFs with greater confidence.

### WGCNA uncovered cell-type-specific gene modules and functions in immune cells

**Figure 2:**
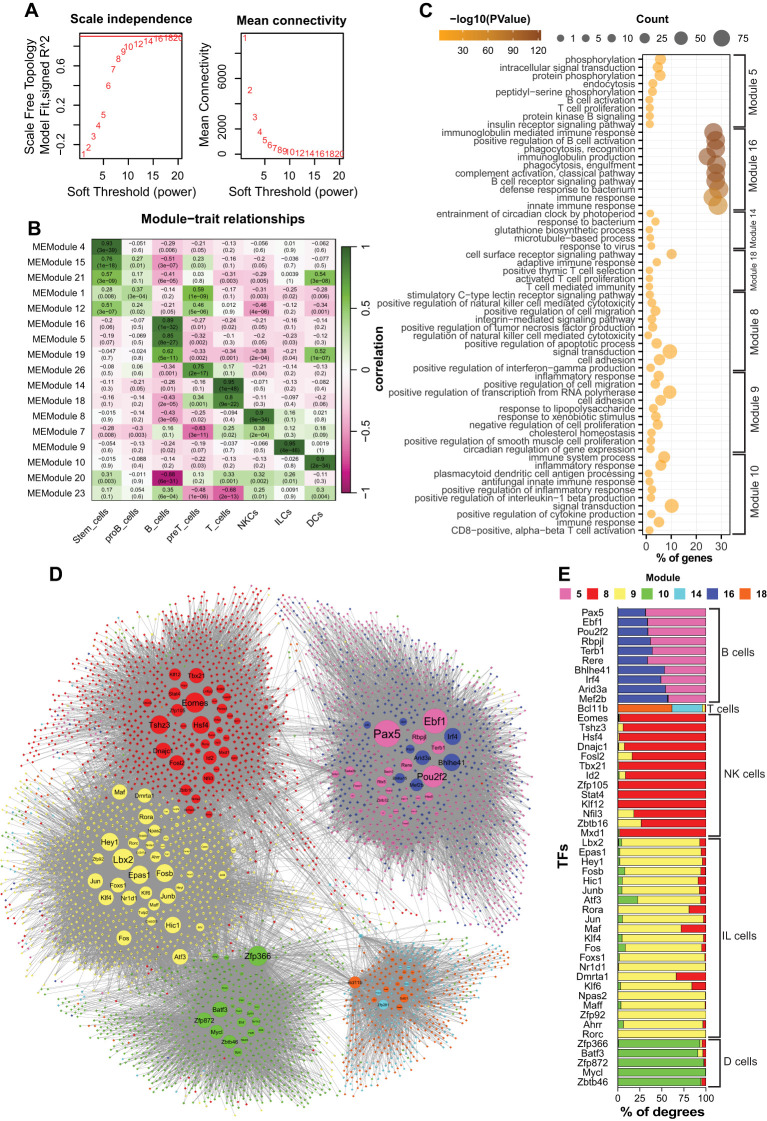
The results of WGCNA and network analysis of the identified modules. **(a)** Analysis of the scale-free topology network of the WGCNA. **(b)** Heatmap showing the relationships between the modules and cell groups. **(c)** GO analysis of the related modules. **(d)** Integrated network of the related modules to the blood cells. Circles are TFs, and rhombi are non-TF genes. Larger nodes have higher connectivity (degree). Purple and dark blue: B cells; cyan and orange: T cells; red: NKCs; yellow: ILCs; Green: DCs. **(e)** Stacked bar chart showing the distribution of connections with genes to different modules for the top 50 most connected TFs. DCs, dendritic cells; ILCs, innate lymphoid cells; NKCs, natural killer cells.

WGCNA constructs weighted gene co-expression networks to identify clusters of co-expressed genes and their associations with specific features across samples (28). We applied this technique to identify genes associated with each cell group. Using a soft power of 16, we identified 23 modules correlating to the cell groups (**Figures 2a, b**). We then focused on lymphoid cells and DCs to further identify the genes associated with each cell type. For each cell type, at least one module with the highest correlation was selected. Since B and T cells were strongly correlated with two modules (16 and 5, and 14 and 18, respectively), we included both sets of modules in the further analysis (**Figure 2b**). Each module comprises a different number of genes, ranging from 121 (Module 26) to 2,654 (Module 1). We excluded proB and preT cells because they either resulted in weakly correlated modules (proB cells) or yielded redundant GO results (preT cells) (data not shown). We then focused on differentiated blood cells that had progressed beyond the stemness stages. To assess the biological relevance of the modules, we performed GO enrichment analysis for the genes constructing each module (**Figure 2c**). The results indicate that the modules were significantly (p-value ≤ 0.01) correlated with the function or development of the corresponding cell types, as expected. For example, modules 5 and 16 were associated with B-cell development (e.g., BCR signaling, antibody production), module 8 was associated with NK cell function (e.g., cytotoxicity, chemokine production), and module 10 was associated with DCs (e.g., inflammatory responses and antigen processing) (**Figure 2c**). All modules showed high levels of statistical significance in the GO analysis (p-value ≤ 0.01). Notably, module 16 showed very low p-values compared to the others, indicating a strong association between the genes in this module and B cell identity (**Figure 2c**).

Altogether, our results showed that WGCNA successfully identified modules correlated with each cell type. We also highlighted that members of each module were associated with cell-specific biological processes, thus indicating the accuracy of our analysis.

### Identifying cell-specific regulatory elements in immune cells

Each module was composed of dozens to hundreds of genes that encode proteins and non-coding RNA molecules involved in different functions. To understand the molecular mechanisms underpinning the formation of each module, we focused our analysis on the TFs to identify regulatory elements associated with the development and/or function of each cell type. To this end, we retrieved selected modules (5,8,9,10,14,16, and 18) from the network constructed by WGCNA and applied a filter to select all the TFs and their associated genes with a correlation value ≥ 0.02 (weight of edge). The result was a network showing intra- and inter-module correlations between the TFs and connected genes (**Figure 2d**). The topology of the network showed a relatively close interaction between the modules of ILCs, NKCs, and DCs, in which ILCs and NKCs shared several TFs, including *Zbtb16*, *Nfil3*, *Dmrta1*, and *Fosl1* (**Figure 2e**). Additionally, **Figures 2d, e** show that the TFs such as *Atf3*, *Fos*, *Batf3*, and *Zfp366* were correlated with ILCs, NKCs, and DCs. Noticeably, in the shared TFs, the majority of the connections were intra-modular. In contrast, the modules associated with B and T cells showed higher isolation. Each of these cell types was associated with two modules (5 and 16 for B cells, and 14 and 18 for T cells) (**Figure 2b**). For each cell type, the members of the two modules were indistinguishably connected so that the TFs were highly connected to the members of both modules. For example, the *Bhlhe41*, *Irf4*, and *Mef2b* connections were identically shared between modules 5 and 16 (**Figure 2e**). Similarly, *Bcl11b* was connected to modules 14 and 18 associated with T cells (**Figure 2e**).

In conclusion, our results revealed that ILCs, NKCs, and DCs exhibited partially overlapping co-expression networks and shared several TFs. Nonetheless, ILCs and NKCs showed closer association with each other compared to their relationships with DCs, which is in line with their respective reported origin in the bone marrow.

### Identifying cell-specific transcription factors in immune cells through integrated genomic analysis

**Figure 3:**
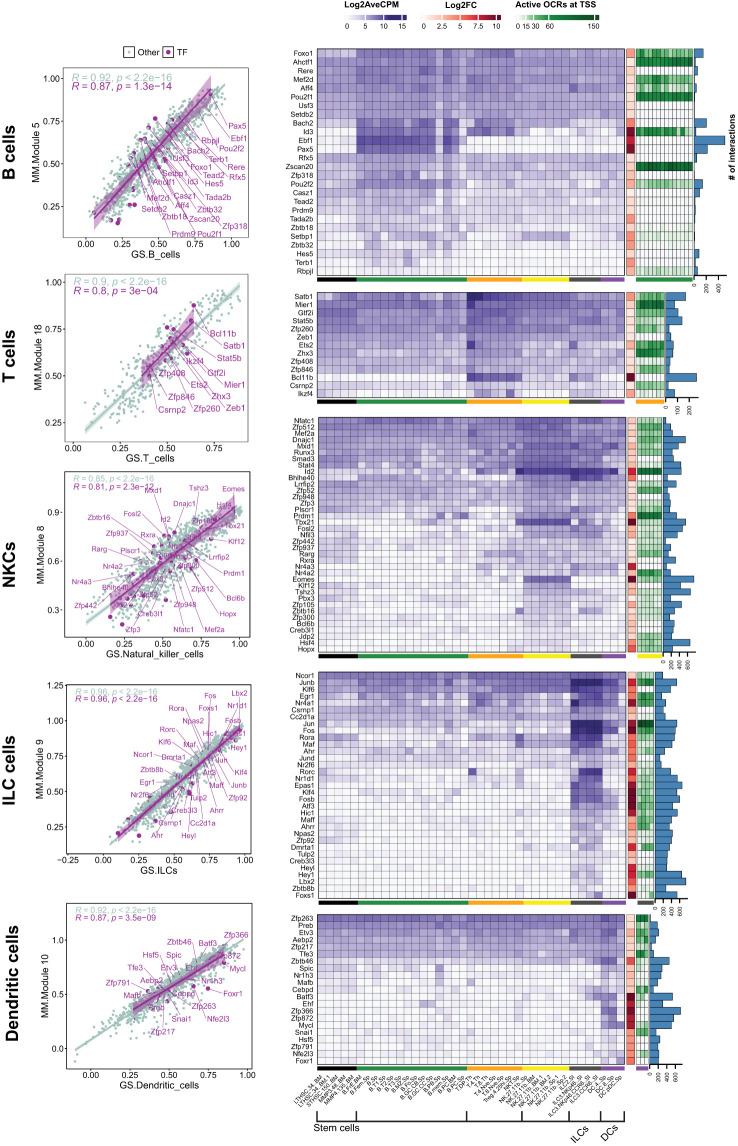
Analysis of TFs in highly related modules to the white blood cells. Scatter plots: The scatter plot shows the correlation of module membership (MM) and gene significance (GS) of the selected modules. The correlations are shown for TFs and genes that belong to other types. Only TFs with an MM ≥ 0.5 are shown. Heatmaps and bar charts: The purple heatmaps show the average gene expression in log2 CPM for selected TFs (MM ≥ 0.05). The orange heatmap shows the changes (Log2FC) in gene expression of corresponding cell families compared to stem cells. A complete list of DEGs in all cell groups is presented in **Supplementary File 3**, **Supplementary Figure S6**. Green heatmaps show the OCR activity at the TSS site of the genes in corresponding cells. Blue columns show the degree number of each TF, indicating the number of connections with other genes within the module. Black: Stem cells; Green: B cells; Orange: T cells; Yellow: NKCs; dark gray: ILCs; purple: DCs.

To identify cell-type-specific TFs, we examined the identified modules to pinpoint candidates that were either exclusively expressed or expressed at a high level in the corresponding cells. Moreover, the candidate TFs must hold a strong position in their modules, which was defined by the module membership (MM) score obtained from WGCNA results. The correlation between MMs and cell types is shown in the scatterplots in **Figure 3**. The scatterplots show TFs and other genes, with corresponding regression lines (*R*) when applicable. We selected TFs with MM ≥ 0.5 to encompass a range of specific (a higher MM score) and more general (a lesser MM score) factors.

In the subsequent step, we characterized candidate TFs by integrating gene expression patterns, chromatin accessibility, and their centrality within the modules. We first visualized the expression levels (Log2CPM) and compared these expression levels across all cell types, which provided insights into the cell-specificity of the candidate TFs (**Figure 3**, purple heatmap). Additionally, we incorporated the expression changes in each cell type relative to stem cells using Log2FC (**Figure 3**, brown heatmap). We then assessed the accessibility of potential cis-regulatory elements at the transcription start site (TSS) of the candidate TFs by incorporating ATAC-seq data specific to corresponding cell types (**Figure 3**, green heatmap). Finally, we queried the intramodular importance of the TFs by analyzing the hubness of the TF in the module, using the degree centrality index, which quantifies the number of connections between a node and all the other nodes within the network (**Figure 3**, bar chart).

Our results showed that the number and specificity of TF expression can vary greatly amongst cell types. Globally, B and T cells showed a limited number of cell-specific TFs compared to the other immune cells analyzed in our study. For example, Ebf1 and Pax5 were two TFs restricted to B cells, while ILCs exhibited a high expression of several TFs, including *Rorc, Jun, Nr1d1, Fosb, Hic1*, and *Epas1* (**Figure 3**). Interestingly, many of the identified TFs were also expressed across multiple cell types. For example, *Id2* was expressed in NKCs and ILCs, and *Pou2f2* was expressed across all cell types, peaking in B cells (**Figure 3**). These findings suggest that while TFs can function in multiple cell types, their impact may be more pronounced in specific groups, depending on the presence of co-interactors that may play crucial roles in modulating their activity/functionality in each cell type. Concordantly, the majority of candidate TFs were also differentially expressed compared to stem cells. Unexpectedly, TFs with cell-specific expression did not always have open chromatin regions (OCRs) at their TSS in the cell type where they were selectively expressed. For example, *Pax5* and *Ebf1* in B cells, *Bcl11b* in T cells, and *Zfp872 i*n DCs had no OCRs at TSS (**Figure 3**). Finally, the centrality of the TFs in modules was strongly correlated with the cell-specificity of the expression. For example, *Epas1* and *Lbx2*, which exhibited relatively high and low expression in ILCs, respectively, and almost no expression in the other cells, were each connected to over 600 genes. In contrast, the TFs that were expressed in multiple cell types, such as Jun and Fos in ILCs, demonstrated reduced centrality. These findings indicate that the centrality of a TF is associated with the specificity of its expression within a particular cell type.

Altogether, these results have identified multiple cell-specific TFs in white blood cells. We also showed that, in some cases, their expression can be dictated independently of the accessibility of their chromatin at their TSS.

### Regulatory networks controlling lymphocyte development

**Figure 4:**
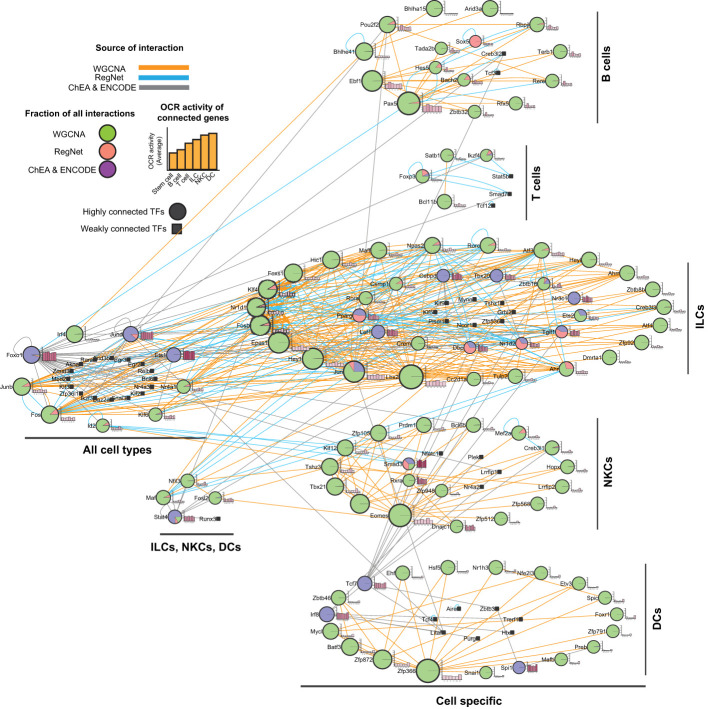
Integrated regulatory network of white blood cells. By combining WGCNA with the RegNet and ChEA2022 datasets, we constructed a large network of gene interactions between TFs and their non-TF interactors and enriched it with the gene expression data to identify cell-specific members expressed in a specific or a group of cells. The resulting network was analyzed for centrality parameters. Next, we constructed a smaller network of only TFs interacting with each other. The TF centrality calculated in the first network was presented as different in the node size. For each TF, the average activity OCR of its targets across all cell families was calculated and presented as a bar chart next to the nodes. Larger nodes indicate a higher degree. The color of the edges shows the type of interaction between the nodes. The color(s) of the nodes show the fraction of edges identified in the datasets. Abbreviations: DCs, dendritic cells; ILCs, innate lymphoid cells; NKCs, natural killer cells.

The distinct patterns of TF expression across blood cells suggest the existence of complex regulatory networks. To better define these networks, we integrated our WGCNA results with data from the RegNetwork (32) and ChEA (31) databases. This approach enabled us to construct a network that integrated multiple aspects of interactions between TFs and other non-TF encoding genes. This includes co-expression, protein-gene, and protein-protein interactions. In addition to the TFs identified through the WGCNA, we included TFs from the ChEA database that are known to regulate genes expressed in each cell type, and any other TFs listed on the RegNetwork database. RegNetwork contains gene regulatory networks based on TF, protein-protein interaction, and microRNA information, which are collected and integrated from 25 selected databases (32). The ChEA database is a compilation of chromatin immunoprecipitation (ChIP) studies that identify TF-targeted genes across a range of cell types (31). In addition to gene clustering derived from the WGCNA results, we also categorized TFs based on their expression level changes compared to the stem cells. Accordingly, TFs could be differentially expressed in a specific (Pax5 in B cells) or multiple cell lineages (Foxo1 and Junb in all cell types). Based on this approach, TFs expressed in multiple cell types were defined as general TFs, either for ILCs, NKCs, and DCs together or all other cells, despite belonging to a specific cell type identified by the WGCNA (**Figure 4**). The resulting network had 3,051 nodes and 15,072 edges (**Supplementary File 3**, **Supplementary Figures S2**, **S3**, respectively). We analyzed this network and extracted information, such as the centrality of the TFs, the type of connections (WGCNA, ChEA, and RegNet), and the cell group(s), to construct a new network restricted to the TFs (**Figure 4**). This network had 170 nodes and 467 edges (**Supplementary File 3**, **Supplementary Figure S4**, **S5**, respectively). Finally, chromatin status was determined for each connected gene and included as a bar chart showing the average OCR activities across all cell types. This visualization provides a comprehensive overview of the regulatory potential of each TF in different cellular contexts.

Using our network, one can predict the significance of TFs in governing blood cell development through factors such as connection type, centrality, specificity, and the activity of their interactors within OCRs. The network shows that the WGCNA result is the dominant indicator of the network topology, particularly in individual cell types. In each module, the majority of the TF connections were based on the expression correlation. Hence, having interactions from other sources can enrich WGCNA results. We must flag that TFs within a cluster may have no direct association. For example, there was no detected interaction between Bcl6b and Mef2a in NKCs. In addition, the number of connections showed the importance of the TF because larger nodes have a higher potential impact on the development. For example, *Pax5*, *Eomes*, and *Foxp3* are known to be important in B cells, NKCs, and regulatory T cells development, respectively (34–36). Specificity is another critical feature for the importance of a TF. Based on the WGCNA results, we identified several cell-specific TFs in each cell type. Remarkably, some of these TFs, i.e., *Foxo1*, *Irf4*, *Jund*, *Junb*, and *Fos*, were expressed in multiple cell types, suggesting their involvement in controlling the development and/or function of these cells, as previously reported. For example, FOXO1 regulates B cells, T cells, and DCs (37–40). JunB is also involved in the development and function of DCs and NK cells (41–43). The differential expression of some TFs compared to stem cells, coupled with their shared expression across a range of differentiated immune cells, suggests that these TFs may have critical roles in controlling the development and function of these immune cells, which warrants further investigation.

Finally, we showed that the non-TF encoding genes that interact (protein-gene interaction or gene co-expression) with the TFs generally have more active OCRs at TSS. For example, the genes connected to *Pax5*, *Ebf1*, and *Pou2f2*, possess higher active OCRs in B cells compared to other cells (**Figure 4**). These results indicate a direct correlation between TFs and the accessibility of cis-regulatory elements of their associated genes.

Altogether, our network analysis underscores the importance of several TFs in governing white blood cell development and/or function. Additionally, our analysis reveals the interaction and potential roles of TFs within a particular subset of cells or across multiple immune cells. These findings offer valuable insights to study the regulatory mechanisms controlling white blood cell development.

### Identification and prioritization strategies for novel transcription factor candidates regulating immune cell development and function

Our analysis identified a range of TFs involved in hematopoiesis, including both well-established TFs and potentially novel candidates. To extract from our analysis the TFs with an unknown function in controlling immune cell development, we conducted a comprehensive literature review, focusing on the TF names and their associated cell families. We classified TFs as known if they have been mechanistically or experimentally validated in relation to blood cell development or function. Using this strategy, we identified 71 novel TF candidates, whose function in the expressing cells remains to be elucidated (**Tables 1**, **2**). Among all the TFs, 78 (> 50% of all identified TFs) have already been reported to control immune cell development or function (**Supplementary File 3**, **Supplementary Figure S1**). Interestingly, all the TFs associated with the T cell lineage were known with reported functions, in contrast to the ILC and NK lineages, which contain several TFs whose functions remain to be addressed (**Supplementary File 3**, **Supplementary Figure S1**). Additionally, our analysis revealed that ubiquitous TFs (found across immune cells) have been mostly experimentally validated with the exception of FOSL2 whose function in ILCs, NKs, and DCs remains unknown.

**Table 1:** The identified TFs in this study with no reported function.

Cell group	Gene symbol ^*^	Expression in corresponding cell (Log2 CPM)	Log2FC (vs Stem cells)	Specificity	Degree	Network types	OCR activity of interactors
**B cells**	Bhlha15	2.98	6.55	2.35	189	WGCNA	1.50
	Rbpjl	1.04	2.92	2.93	118	RegNet	8.48
	Rere	7.26	1.78	1.16	73	RegNet_WGCNA	7.12
	Tada2b	3.41	4.04	1.46	27	WGCNA	7.14
	Terb1	1.98	4.06	3.22	98	WGCNA	8.34
**NKCs**	Bcl6b	3.16	2.45	2.82	123	chea	9.27
	Creb3l1	1.95	1.96	1.80	99	chea	8.41
	Dnajc1	7.37	1.14	1.12	40	WGCNA	21.15
	Hopx	1.85	5.99	2.87	89	RegNet_WGCNA	7.51
	Hsf4	2.66	4.25	3.24	310	WGCNA	10.90
	Mef2a	8.05	1.99	1.16	108	RegNet_WGCNA	8.15
	Rxra	4.38	1.27	1.48	9	WGCNA	19.35
	Tshz3	4.78	2.79	2.07	217	WGCNA	12.12
	Zfp105	3.90	3.32	2.42	179	WGCNA	10.60
	Zfp512	8.25	1.37	1.19	55	WGCNA	6.79
	Zfp568	5.01	1.40	1.26	75	WGCNA	7.87
	Zfp948	5.91	1.36	1.28	39	WGCNA	8.07
**ILCs**	Ahrr	4.71	4.16	2.86	137	WGCNA	8.04
	Atf4	8.41	1.68	1.12	124	WGCNA	6.47
	Cc2d1a	6.24	2.20	1.28	76	WGCNA	6.84
	Cebpd	3.44	2.10	1.78	45	chea	25.61
	Creb3l3	2.02	2.08	3.06	126	RegNet_WGCNA	7.10
	Crem	6.65	3.60	1.36	76	WGCNA	6.20
	Csrnp1	7.23	3.14	1.39	56	RegNet_WGCNA	11.46
	Dmrta1	3.79	7.33	3.42	124	WGCNA	5.83
	Epas1	8.23	9.03	3.81	402	WGCNA	10.59
	Ets2	4.88	1.73	1.05	21	chea	9.63
	Fosb	8.66	10.03	3.41	355	RegNet_WGCNA	10.09
	Foxs1	3.51	10.03	4.93	280	WGCNA	8.15
	Hey1	3.93	7.40	5.21	405	chea	10.66
	Heyl	3.35	7.38	4.08	158	RegNet_WGCNA	7.40
	Jun	12.38	8.93	2.33	408	chea	11.62
	Klf4	8.31	10.02	3.45	300	RegNet_WGCNA	10.49
	Lbx2	3.14	5.98	5.61	528	RegNet	11.40
	Npas2	3.95	2.86	2.71	187	chea	8.56
	Pparg	2.18	3.25	1.69	64	chea	20.02
	Tbx20	0.94	3.37	1.52	41	chea	16.83
	Tgif1	7.58	1.12	1.27	17	chea	18.60
	Tulp2	2.79	2.96	2.35	117	WGCNA	8.74
	Zbtb16	4.48	2.94	2.06	27	chea	13.32
	Zbtb8b	1.71	4.04	2.99	132	WGCNA	5.64
	Zfp92	4.46	2.47	3.49	124	WGCNA	7.44
**DCs**	Aebp2	6.42	1.83	1.10	14	WGCNA	3.11
	Ehf	3.92	8.15	3.37	104	WGCNA	5.27
	Foxr1	3.63	5.21	4.55	49	WGCNA	11.47
	Hsf5	2.18	3.96	2.52	100	WGCNA	5.41
	Litaf	7.93	2.08	1.35	5	chea	38.92
	Nfe2l3	3.17	4.34	2.78	63	WGCNA	7.88
	Nr1h3	3.74	2.68	2.27	91	WGCNA	7.81
	Preb	7.52	1.10	1.05	39	chea	6.28
	Purg	1.81	1.53	1.53	1	chea	33.50
	Snai1	1.97	4.02	2.32	23	WGCNA	4.65
	Spic	4.93	2.12	2.55	56	WGCNA	10.17
	Tcf7	2.73	3.88	0.59	120	chea	18.88
	Trerf1	6.52	1.91	1.26	2	chea	27.95
	Zbtb3	2.86	1.15	1.44	2	chea	20.45
	Zfp791	1.20	4.18	2.09	41	WGCNA	5.17
	Zfp872	4.18	8.30	3.78	305	WGCNA	8.46

^*^ The gene symbols are sorted alphabetically.

**Table 2:** The identified TFs associated with DC lineage development with no reported function.

Cell group	Gene symbol^*^	Expression in DC4s (Log2 CPM)	Expression in DC8s (Log2 CPM)	Expression in pDCs (Log2 CPM)	Specificity
**DC4s**	E2f2	5.88	2.82	0.09	0.83
	Trps1	8.38	6.64	5.01	1.15
	Zfp263	9.43	8.39	8.01	1.08
**DC8s**	Atf3	6.18	5.64	0.35	2.02
	Ehf	6.55	4.95	0.25	3.37
	Fosb	5.45	5.28	1.01	1.54
**pDCs**	Bhlha15	0.17	0.89	5.41	1.70
	Cdip1	6.00	6.01	9.00	1.04
	Gcm2	0.00	0.08	3.69	2.85
	Hivep3	3.76	3.71	7.41	1.01
	Sp100	7.23	6.81	8.35	0.93
	Zfp658	3.18	1.20	7.04	1.20
	Zfp810	6.06	5.97	7.86	1.10

^*^The gene symbols are sorted alphabetically.

To prioritize the study of unknown TFs, we employed strategies based on expression criteria such as expression levels, fold changes, and cell-specificity. Network centrality metrics were used to rank the TFs, with a higher number of connections indicating greater hubness in the regulatory network (44). Additionally, we integrated data from multiple sources (WGCNA, ChEA, and RegNet) to ensure robust identification. We also used the average OCR activity of TF-associated genes as a sorting criterion, where a higher average suggests a link between TF activity and the chromatin accessibility of its target genes (**Supplementary File 3**, **Supplementary Figure S4**).

Overall, our analysis validates the effectiveness of our strategy by confirming a substantial number of TFs previously known to regulate the development and function of immune cells. Importantly, our approach also uncovers multiple novel TFs with potential roles in these processes. By employing innovative sorting criteria, we enhanced the identification and prioritization of novel TFs involved in white blood cell regulation. To further validate this strategy, we will conduct a case study where *in silico* analysis leads to the identification of a new TF regulating cell fate decisions.

### Deciphering dendritic cell lineage by unveiling transcription factor network

**Figure 5:**
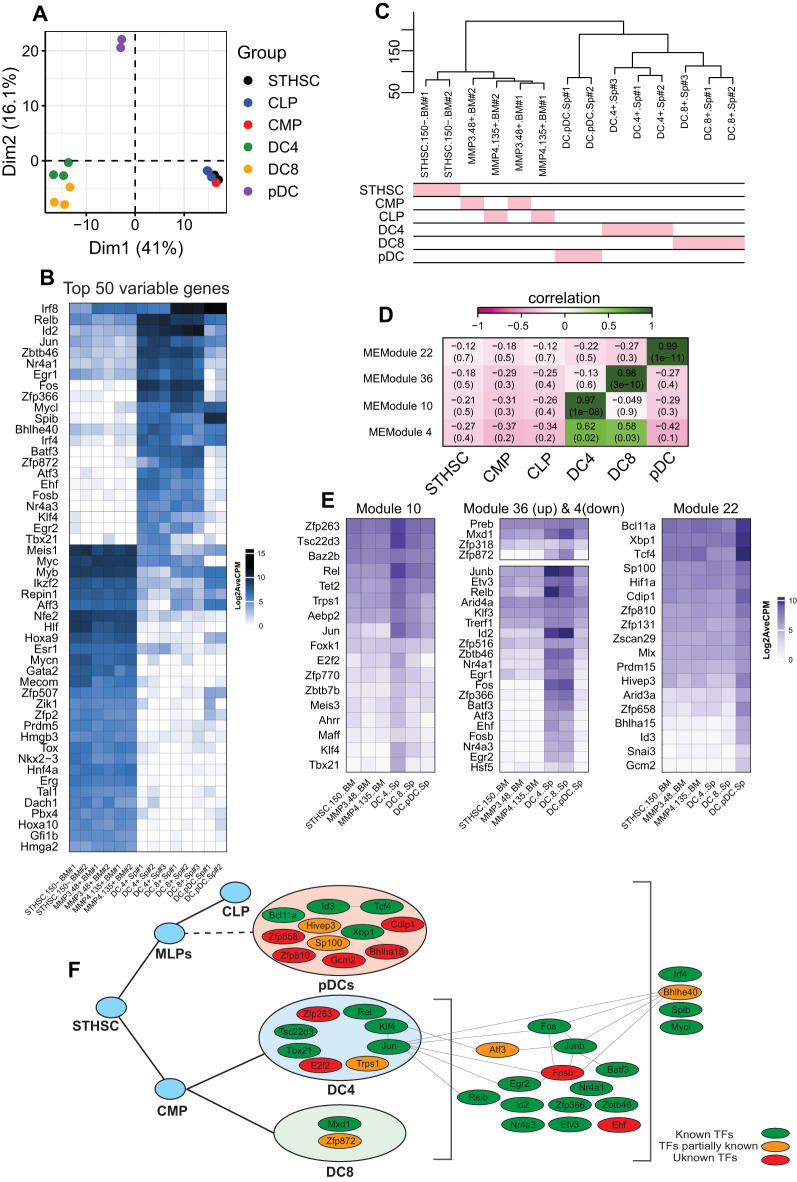
Analysis of DCs with WGCNA and association of TFs with each cell type. **(a)** PCA plot of stem cells and DCs. **(b)** Heatmap of the top 50 most variable genes in stem cells and DCs. **(d)** Hierarchical clustering of stem cells and DCs. **(d)** Heatmap of the module and cell relationships of only correlated modules. **(e)** Heatmaps of the gene expression of TFs with module membership (MM) ≥ 0.5. **(f)** The map of TFs expressed in DCs and their identified roles in DC development and/or function.

DCs are antigen-presenting cells with the ability to orchestrate adaptive immune responses (45, 46). Despite their pivotal role in shaping immune surveillance, a comprehensive understanding of their origin and functional specialization remains a matter of debate (47, 48). According to the established paradigm of DC specification, myeloid-primed progenitors give rise to both conventional DC subsets (cDCs: type-1 or type-2 cDCs) and plasmacytoid DCs (pDCs). Lineage specification into these functionally distinct subsets is tightly regulated by transcription factors. For instance, BATF3 and IRF8 are essential for cDC1 differentiation (49, 50), while IRF4, NOTCH2, and KLF4 play a role in defining cDC2 identity (51–54). Importantly, ID2, a key inhibitor of E protein activity, acts by repressing E2.2 (*Tcf4*) activity in progenitors, which is essential for the development of pDC (55). This inhibition allows the initiation of the cDC program in progenitors and the subsequent development of cDC1 and cDC2 subsets (16, 17, 45). This traditional view serves as the foundation for uncovering the molecular mechanisms that govern DC lineage specification (17, 56). However, recent studies have challenged this paradigm by disputing the myeloid origin of pDCs, proposing instead that pDCs predominantly originate from a lymphoid-primed progenitor (57–59). Consistent with this emerging view, our findings indicate that, although cDCs and pDCs cluster together, their expression of TFs vastly differs (**Figure 3**). Therefore, we performed a separate WGCNA analysis for DCs, including stem cells, cDC2s (DC4s), cDC1s (DC8s), and pDCs (**Figure 5**). Our results showed that stem cells (STHSC, CMP, and CLP) were closely clustered and cDC2s and CDC1s were grouped together in a separate cluster. In contrast, pDCs formed a distinct cluster apart from stem cells and cDCs (**Figures 1A, C**), which is reflective of significant differences between conventional and plasmacytoid DCs transcriptomes (22). We examined the 50 most variable genes in the samples and identified several candidates across cell types. For example, *Relb*, *Id2*, *Fos*, and *Zfp366* were expressed only in cDC2s and cDC1s, not pDCs (**Figure 5b**), whereas *Spib* was selectively expressed in pDCs (60). Conversely, the WGCNA results showed multiple specific genes for DC types through four highly correlated modules (**Figure 5d**). We visualized highly correlated TFs (MM > 0.8) in each module to compare their expression levels across cDC2cs, cDC1s, and pDCs (**Figure 5e**). We found several TFs that were uniquely correlated with cDC2s (module 10), including *Zfp263*, *Tsc22d3*, and *Rel* (**Figure 5e**). In contrast, *Mxd1* and *Zfp872* showed moderate correlation with cDC1s, despite also being slightly expressed by cDC2s. We also searched module 4, which was associated with both cDC2s and cDC1s, and identified several highly expressed TFs, such as *Junb*, *Relb*, *Id2*, *Fos*, and *Zfp366* (**Figure 5e**). Finally, we found multiple TFs highly expressed in pDCs, including the known transcription factors *Tcf4*, *Bcl11a*, and *Xbp1*, but also ill-studied TFs such as *Zfp658*, *Cdip1*, *Bhlha15*, and *Hivep3*.

Based on these results, we constructed a regulatory logic of TFs that potentially regulate the DC development (**Figure 5f**). In this framework, TFs were categorized into pDC, cDC-specific, and general factors that regulate all DCs. Our analysis revealed a few known interactions among these TFs. Although a literature review identified several of these TFs (green nodes) as established key regulators of DC development and function, many candidates (orange and red nodes) remain to be investigated further (**Figure 5d**, **Tables 1**, **2**).

### BHLHA15 (Mist1) inhibits conventional dendritic cell differentiation

Our data identified several TFs with poorly defined roles in DC differentiation and function (**Figure 5d**). Among these, BHLHA15 (also known as Mist1) emerged as a prime candidate for further investigation. BHLHA15 efficiently binds to E-box sequences as a homodimer but can also bind as a heterodimer with E-proteins (61). Importantly, the latter, particularly E2.2, is crucial in determining the lineage decision between pDCs and cDCs from progenitor cells (55, 56). This suggests that BHLHA15 may play a significant role in regulating DC lineage fate decisions.

**Figure 6:**
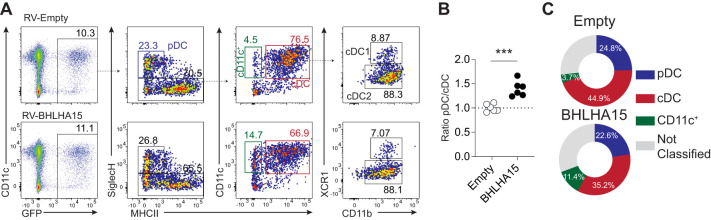
Experimental validation of candidate TFs. **(A)** Flow cytometric analysis of bone marrow-derived DCs transduced with either GFP encoding (empty: top row) or BHLHL A 15 viruses (bottom row). **(B)** The graph represents the ratio of pDC to cDC (normalized to empty control) of BMDC transduced with the control or BHLHA 15 viruses. The results are shown from two independent experiments (n=3/exp). **(C)** Pie charts represent the frequency of pDC, cDCs (CD11c^+^ MHC-II^+^), and CD11c^+^ (MHC-II^-^) cells in the BMDCs that were successfully retrotransduced (GFP^+^) with empty (control) or BHLHA15-encoding viruses. Data shown are representative of two independent experiments (n=3/exp).

To test this hypothesis, bone marrow progenitors were cultured in the presence of Flt3L and retrovirally transduced with either a control virus encoding GFP or a virus encoding BHLHA15 (**Figure 6A**). Five days post-transduction, the differentiation of bone marrow-derived DCs was analyzed by flow cytometry. While all three subsets, i.e., pDCs (pDCs; SiglecH^+^, MHCII^-/low^), cDC1s (cDC1s; SiglecH^-^, CD11c^+^ MHCII^+^, XCR1^+^), and cDC2s (cDC2s; SiglecH^-^, CD11c^+^ MHCII^+^, XCR1^-^), were generated from progenitors transduced with either the control or BHLHA15-encoding virus, the overexpression of BHLHA15 significantly impaired the differentiation into conventional DCs (**Figures 6A, B**). Additionally, we noted a substantial accumulation of immature CD11c^+^ MHCII^-^ cells upon BHLHA15 retrotransduction, indicative that BHLHA15 may prevent the differentiation of progenitors into cDCs (**Figure 6C** and data not shown). These findings suggest that BHLHA15 may function as a negative regulator of cDC differentiation.

## Discussion

A fundamental requirement for a multicellular organism is the establishment of an extensive network of specialized cells, each with unique functions that collaboratively contribute to the development and function of tissues and organs. The hematopoietic system provides a perfect illustration of this concept. For example, multipotent hematopoietic stem cells in the bone marrow undergo differentiation into functionally distinct lineages whose specialization is paramount for vital processes such as oxygen transport, immune surveillance, and hemostasis. As the development of a functional immune system requires dynamic regulation of transcription factor networks that activate lineage-specific gene expression and restrict the differentiation options of hematopoietic progenitors, we reasoned that a description of both the wiring and the logic of these transcriptional networks is essential for a complete understanding of immune cell development. Our study leveraged transcriptomic data from highly purified immune cells provided by the ImmGen project, which is well-suited for systems-level analysis. By integrating ATAC-seq data and applying WGCNA, we constructed a network of co-expressed genes and further enriched it with gene-protein interaction data. This approach enabled us to identify key regulatory circuits and hub TFs associated with the development and function of immune cells.

Network-based analyses have proven effective in uncovering hub genes and proteins across various biological systems (62–67). Previous studies have investigated regulatory networks in blood cell development, including TF gene regulatory interactions in intestinal ILCs (63) and distinct TF expression patterns in stem and progenitor cells (64). However, these studies often concentrated on specific cell types or HSCs. Although a previous study identified the transcriptional regulatory networks for various mouse immune cell types (65), to date, most network-based analyses have either focused on HSCs or specific immune cell types. In our study, we applied WGCNA to identify co-expressed genes uniquely associated with each cell family, providing a detailed network analysis (28). Our approach benefits from an unbiased analysis that leverages gene expression variability among samples, allowing us to capture insights beyond those provided just by DEGs (68). Additionally, we not only identified highly expressed and cell-specific TFs but also characterized subtle, yet cell-specific, changes in the transcriptome profile. We then enriched the WGCNA results with gene-protein and protein-protein interactions, and DEGs, to construct a blueprint regulatory network controlling lymphoid cells and DCs development and function. In addition, applying ATAC-seq demonstrated higher activity of cis-regulatory elements of the TF-associated genes in each group (22). Altogether, our multi-layered blueprint network highlights central TFs regulating different cell lineages and their functional specificity or redundancy in blood cell development. However, our current network lacks DNA sequence motifs for TF-gene interactions, which would enhance the accuracy of the regulatory network.

Multiple TFs have been identified that regulate blood cell development. The functionality of TFs could be restricted to a specific lineage, such as PAX5 and EBF1 in B cells (14, 15). In contrast, other TFs, such as FOXO1, have broader roles and influence multiple lineages, including B cells, T cells, and dendritic cells (37–40). Our study uncovered several TFs with potential roles in lymphoid cells and DC development and/or function. We combined all related sub-populations as a single lineage due to their close relationships. Notably, proB and preT cells were excluded from analysis of the corresponding matured cells because their characteristics are more closely aligned with those of stem cells. We showed that the expression of most of the identified lineage-specific TFs was highly consistent across all the sub-groups and showed high statistically significant differential expressions compared to stem cells. These indicate a potential functionality in cell development and/or function. Noticeably, the lack of chromatin accessibility at the TSS of some of these TFs suggests the existence of regulatory mechanisms through distal-regulatory elements. However, attempts to identify these distal regulatory elements could be compromised by the presence of multiple genes within the 100kb region from the TSS, although integration of chromatin accessibility with expression of nearby genes in the relevant cells may help determine if distal regulatory elements are more likely associated with distantly expressed genes rather than nearby non-expressed genes (22).

By performing a separate WGCNA analysis specifically for DCs, our study revealed that cDCs and pDCs possess a set of exclusive regulatory TFs. It further evidenced the central role played by transcription factors such as IRF8, PU.1 (encoded by *Spi1)* and DC-SCRIPT (encoded by *Zfp366)* in controlling cDC identity (18, 19, 69–71). Interestingly, we identified a substantially higher number of transcription factors associated with cDC2s compared to cDC1s, suggesting not only their functional differences but also that cDC2s may represent a more heterogeneous population of DCs, in contrast to the relatively well-characterized cDC1s in terms of ontogeny and function (17, 45, 46, 72). Importantly, our *in silico* analysis identified previously uncharacterized transcription factors associated with DC lineage commitment. Among these transcription factors, we demonstrated through a gain-of-function approach that the basic helix-loop-helix transcription factor BHLHA15 plays a regulatory role in progenitor cell fate decisions by inhibiting differentiation into cDCs, although its overexpression did not affect the subsequent specification of cDC1 vs. cDC2 subsets. These results suggest that BHLHA15 acts upstream of cDC lineage bifurcation, most likely influencing the decision made by the progenitor to commit to either pre-conventional DCs or pre-plasmacytoid DCs. Our results further support previous studies derived from murine models suggesting that pDC and cDC lineage specification occurs within the bone marrow, rather than being determined by signals from peripheral tissues (49, 73). Although the molecular mechanisms by which BHLHA15 inhibits cDC specification remain to be fully elucidated, we posit that BHLHA15 expression in progenitors competes with ID2 for E protein binding (61). This competition could prevent ID2-mediated inhibition of E2.2, thereby blocking the differentiation into conventional dendritic cells (cDCs) (55, 74).

## Conclusion

We developed a comprehensive regulatory network that elucidates the control mechanisms underlying the development and function of immune cells. This network serves as a strategic blueprint to guide future research endeavors to decipher the role of newly identified transcription factors in the formation of the immune system.

## Data Availability

The original contributions presented in the study are included in the article/**Supplementary Material**. Further inquiries can be directed to the corresponding authors.
